# Predictive factors for cosmetic surgery: a hospital-based investigation

**DOI:** 10.1186/s40064-016-3188-z

**Published:** 2016-09-13

**Authors:** Jun Li, Qian Li, Bei Zhou, Yanli Gao, Jiehua Ma, Jingyun Li

**Affiliations:** 1 State Key Laboratory of Reproductive Medicine, Department of Plastic and Cosmetic Surgery, Nanjing Maternity and Child Health Care Hospital Affiliated to Nanjing Medical University, 123rd Tianfei Street, Mochou Road, Nanjing, 210004 China; 2 State Key Laboratory of Reproductive Medicine, Department of Reproductive Health, Nanjing Maternity and Child Health Care Hospital Affiliated to Nanjing Medical University, 123rd Tianfei Street, Mochou Road, Nanjing, 210004 China

**Keywords:** Predictive factors, Cosmetic surgery, Eye surgery, Botox injection, Nevus removal

## Abstract

**Background:**

Cosmetic surgery is becoming increasingly popular in China. However, reports on the predictive factors for cosmetic surgery in Chinese individuals are scarce in the literature.

**Methods:**

We retrospectively analyzed 4550 cosmetic surgeries performed from January 2010 to December 2014 at a single center in China. Data collection included patient demographics and type of cosmetic surgery. Predictive factors were age, sex, marital status, occupational status, educational degree, and having had children. Predictive factors for the three major cosmetic surgeries were determined using a logistic regression analysis.

**Results:**

Patients aged 19–34 years accounted for the most popular surgical procedures (76.9 %). The most commonly requested procedures were eye surgery, Botox injection, and nevus removal. Logistic regression analysis showed that higher education level (college, *P* = 0.01, OR 1.21) was predictive for eye surgery. Age (19–34 years, *P* = 0.00, OR 33.39; 35–50, *P* = 0.00, OR 31.34; ≥51, *P* = 0.00, OR 16.42), female sex (*P* = 0.00, OR 9.19), employment (service occupations, *P* = 0.00, OR 2.31; non-service occupations, *P* = 0.00, OR 1.76), and higher education level (college, *P* = 0.00, OR 1.39) were independent predictive factors for Botox injection. Married status (*P* = 0.00, OR 1.57), employment (non-service occupations, *P* = 0.00, OR 1.50), higher education level (masters, *P* = 0.00, OR 6.61), and having children (*P* = 0.00, OR 1.45) were independent predictive factors for nevus removal.

**Conclusions:**

The principal three cosmetic surgeries (eye surgery, Botox injection, and nevus removal) were associated with multiple variables. Patients employed in non-service occupations were more inclined to undergo Botox injection and nevus removal.

**Level of evidence:**

Cohort study, Level III.

## Background

A “normal” appearance is considered important for social interaction and activities (Kim et al. 2013). Until recently, aesthetic surgery was performed only on actors or actresses for professional reasons. Today, a growing number of urban and rural residents worldwide aspire to undergo aesthetic surgery. Cosmetic surgery traditionally includes facial plastic surgery (Hassouneh and Brenner 2015) [e.g., blepharoplasty, rhinoplasty, orthognathic surgery (Harrington et al. 2015), and face lifts] and other body contouring procedures (Abbed et al. 2015), such as breast augmentation and abdominoplasty.

The epidemiology of cosmetic surgery has been described in multiple publications, based on the literature (Zhang et al. 2012; Sreekar et al. 2012; Pu 2012; Go et al. 2012) and statistical data from countries like the United States (Broer et al. 2014). In China, cosmetic surgery has been practiced since 1980. A bibliometrics study reported that the number of articles published by Chinese authors has increased markedly since 2004 (Zhang et al. 2012). However, little is known about the predictive factors for cosmetic surgery in China, the country with the world’s largest population. Therefore, we collected retrospective cross-sectional data from our institution to identify predictive factors for the three major cosmetic surgeries (eye surgery, Botox injection, and nevus removal) carried out in China.

## Results

All variables and the corresponding response options are listed in Table 1. A total of 4550 patients were included. Patients aged 19–34 years accounted for the most popular surgical procedures (76.9 %). The mean age of respondents was 28.1 ± 7.8 (range 16–66) years (Table 1).

**Table 1:** Patient demographics (N = 4550)

Categorical variables (SPSS information)	N (%)
Age (years)	
≤18 (0)	194 (4.3)
19–34 (1)	3499 (76.9)
35–50 (2)	768 (16.9)
≥51 (3)	89 (1.9)
Sex	
Male (0)	115 (2.5)
Female (1)	4435 (97.5)
Marital status	
Unmarried (0)	2590 (56.9)
Married (1)	1960 (43.1)
Occupational status	
Unemployed (0)	1215 (26.7)
Employed in service industries (1)	2555 (56.2)
Employed in non-service industries (2)	780 (17.1)
Educational degree	
Primary, junior or high school only (0)	1674 (36.8)
College or university (1)	2754 (60.5)
Master’s or further education (2)	122 (2.7)
Having had children	
No (0)	2877 (63.2)
Yes (1)	1673 (36.8)

**Fig. 1:**
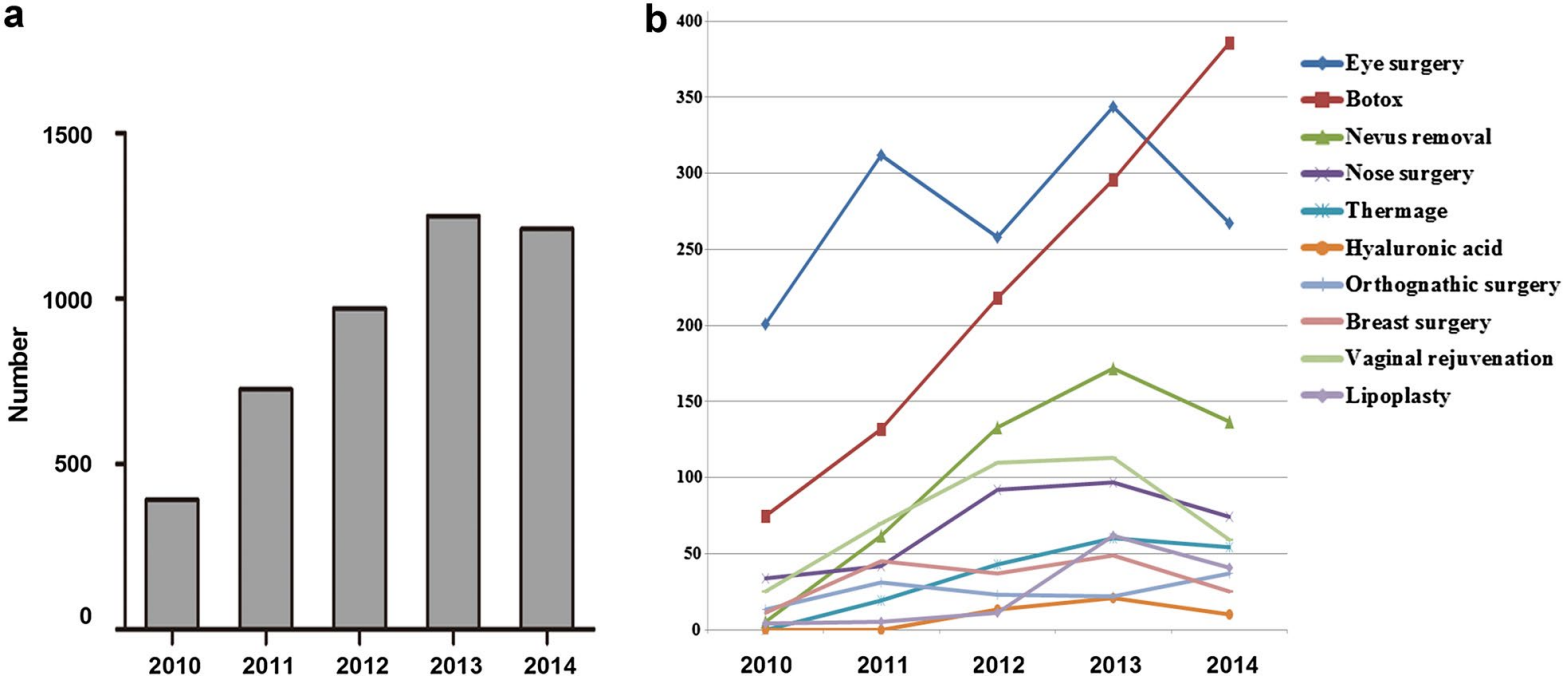
Current trends of cosmetic surgery in our hospital. **a** Number of cosmetic surgeries from 2010 to 2014. **b** Numbers of the leading ten cosmetic surgical procedures from 2010 to 2014

Surgical procedures increased annually, and tripled over a 5-year period (Fig. 1a). The top ten cosmetic surgeries in our hospital are shown in Fig. 1b. Eye surgery represents double eyelid surgery, reconstruction of the inner canthus, eye bag removal, and resection of the levator palpebrae superioris muscle. Nose surgery consists of rhinoplasty augmentation, nose correction, and nasal tip contouring. Botulinum toxin (Botox) treatment constitutes injecting Botox into the masseter muscle, orbicularis oculi muscle, frontal muscle, or leg. Breast surgery comprises breast augmentation, breast reduction, breast reconstruction, and breast lift. Lipoplasty includes abdominoplasty, liposuction, and lipofilling. Vaginal rejuvenation incorporates hymenorrhaphy, colporrhaphy, and nympholepsy. As shown in Fig. 1b, the most commonly requested procedures are eye surgery, Botox injection, and nevus removal. Overall the number of vaginal rejuvenations has escalated sharply, with an average annual growth of 63.2 %. Other striking increases have occurred in nose surgery, thermage, hyaluronic acid treatment, lipoplasty, and breast surgery.

Patients’ age is a possible predictive factor for cosmetic surgery. As shown in Table 2, patients aged 19–34 years accounted for the largest population undergoing all major cosmetic surgeries except thermage and hyaluronic acid.

**Table 2:** Number of cosmetic surgery by age groups

	Number				Total number
Age groups	≤18 (N = 194)	19–34 (N = 3499)	35–50 (N = 768)	≥51 (N = 89)	
Cosmetic surgery					
Eye surgery	92	1134	124	32	1382
Nose surgery	19	286	33	1	339
Orthognathic surgery	14	105	6	1	126
Botox	2	903	189	13	1107
Thermage	0	13	143	20	176
Hyaluronic acid	2	20	22	0	44
Nevus removal	32	417	55	5	509
Breast surgery	0	79	86	2	167
Vaginal rejuvenation	5	326	44	2	377
Lipoplasty	0	90	28	5	123

Logistic regression analysis of independent predictive factors for the top three cosmetic surgeries (eye surgery, Botox injection, and nevus removal) is shown in Table 3. Female patients are inclined to undergo Botox injection (*P* = 0.00, OR 9.19) but do not tend to undergo nevus removal. Married patients are more likely to undergo nevus removal (*P* = 0.00, OR 1.57). Patients employed in the service occupations show a tendency toward Botox injection (*P* = 0.00, OR 2.31). Patients with higher education attainment are inclined to undergo eye surgery (college; *P* = 0.01, OR 1.21) and Botox injection (college; *P* = 0.00, OR 1.39). Patients who have had children show a preference for nevus removal (*P* = 0.00, OR 1.45). Interestingly, all age groups were significantly associated with Botox injection (Table 3).

**Table 3:** Predictor variables for eye surgery, Botox injection and nevus removal: a logistic regression analysis

Categorical variables	Eye surgery				Botox injection				Nevus removal			
	Sig. (*P*)	Adjusted OR	95 % CI		Sig. (*P*)	Adjusted OR	95 % CI		Sig. (*P*)	Adjusted OR	95 % CI	
			Lower	Upper			Lower	Upper			Lower	Upper
Age (years)												
≤18		Referent				Referent				Referent		
19–34	0.00**	0.53	0.40	0.71	0.00**	33.39	8.27	134.76	*0.06*	*0.68*	*0.46*	*1.01*
35–50	0.00**	0.21	0.15	0.30	0.00**	31.34	7.70	127.43	0.00**	0.39	0.24	0.62
≥51	*0.07*	*0.62*	*0.37*	*1.04*	0.00**	16.42	3.62	74.50	0.02*	0.30	0.11	0.80
Sex												
Male		Referent				Referent				Referent		
Female	*0.22*	*1.30*	*0.85*	*1.99*	0.00**	9.19	3.38	24.97	0.00**	0.30	0.20	0.45
Marital status												
Unmarried		Referent				Referent				Referent		
Married	0.00**	0.62	0.54	0.70	0.00**	0.69	0.60	0.79	0.00**	1.57	1.31	1.89
Occupation												
Unemployed		Referent				Referent				Referent		
Service	0.00**	0.46	0.40	0.53	0.00**	2.31	1.93	2.77	0.00**	0.66	0.53	0.82
Non-service	0.00**	0.66	0.54	0.79	0.00**	1.76	1.40	2.22	0.00**	1.50	1.17	1.93
Educational												
Primary		Referent				Referent				Referent		
College	0.01*	1.21	1.06	1.38	0.00**	1.39	1.21	1.61	*0.14*	*1.16*	*0.95*	*1.43*
Master	0.04*	0.62	0.39	0.99	0.01*	0.41	0.22	0.75	0.00**	6.61	4.45	9.83
Had children												
No		Referent				Referent				Referent		
Yes	0.00**	0.70	0.61	0.80	0.00**	0.55	0.47	0.64	0.00**	1.45	1.20	1.74

The OR was adjusted for all variables

*OR* odds ratio, *CI* confidence interval, *NS* not statistically significant

* *P* < 0.05; ** *P* < 0.01

## Discussion

A multitude of factors might motivate patients to request cosmetic surgery, such as psychosocial factors (Nerini et al. 2014), social relationships (Tam et al. 2012), body image (Sarwer et al. 2005), a history of being teased about their appearance (von Soest et al. 2006), and self-esteem (Haas et al. 2008). Media exposure, fashion trends, and beauty magazines correlate with positive attitudes toward cosmetic surgery (Placik and Arkins 2014; Sharp et al. 2014). Our study found that age, sex, marital status, occupational status, educational level, and previous childbirth were significantly associated with the leading three cosmetic surgeries (eye surgery, Botox injection, and nevus removal).

In the United States, the patient group aged 35–50 years requested the most surgical procedures, representing 40 %, whereas patients aged 19–34 years underwent 28 % of all surgical procedures (Broer et al. 2014). In the present study, we found that patients aged 19–34 years underwent the largest share of all cosmetic surgeries, accounting for 76.9 %, indicating a preference for cosmetic surgeries in China among the younger population. One contributing factor may be the popularity of Korean television series in China among the younger Chinese. It is the case that many Korean television stars have undergone cosmetic surgery, leading to a breakdown of previously held stereotypes and an increase in the number of people considering elective cosmetic surgery. Historically, older patients were more likely undergo face lifts to obtain a youthful, natural look (Paul 2011). In our study only 89 patients aged 51 and older underwent cosmetic surgery in the previous 4 years, suggesting that older people in China are much less interested than Americans in cosmetic surgery. Chinese people are bound by a long history of conservatism and traditional beliefs, and may therefore have a relatively negative attitude toward cosmetic surgery.

British females were reported to have a greater likelihood of undergoing cosmetic surgery (Furnham and Levitas 2012). In South Korea, women were more accepting of cosmetic surgery in comparison with male counterparts (Swami et al. 2012). Our results, likewise, show that in China, as in other countries worldwide, women make up the vast majority of cosmetic surgery patients.

The married population has been reported to be just as eager as the unmarried to undergo cosmetic surgery to gain support of family, friends, and partners, and to better their social positions and values, besides making their appearance more attractive to others (Salehahmadi and Rafie 2012). Marital status was reported to be statistically significantly related with Dermatology Life Quality Index scores in vitiligo patients (Dolatshahi et al. 2008). The present study highlighted that marital status was statistically significantly associated with eye surgery, Botox injection, and nevus removal, with a greater tendency toward nevus removal.

A previous study proposed that occupation was a risk factor for hypertensive disorders of pregnancy (Bilhartz and Bilhartz 2013). In the present study, patients employed in the service industries were inclined to undergo Botox injection, perhaps in the belief that improvement in appearance might increase their chances of becoming successful in their career.

Generally speaking, most cosmetic surgery respondents have been educated to undergraduate level (Salehahmadi and Rafie 2012). Our study shows that patients with a higher education level are more likely to undergo eye surgery and Botox injection. We may thus conclude that college students are more aware of the benefits of cosmetic surgery in correcting certain malformations in the face and body and in helping the individual to function better and more comfortably.

In conclusion, the present study offers new insights into demographic factors that affect individuals’ inclination to undergo cosmetic surgery. Plastic surgeons should pay attention to the instructive attributes of predictive factors for cosmetic surgery patients. Even a small cosmetic alteration might be sufficient to give an individual an emotional boost.

## Conclusions

We retrospectively analyzed cosmetic surgeries performed at a single center in China. The study offers new insights into factors influencing the motivation to undergo cosmetic surgery. It is suggested that these factors be included in future research designs pertaining to cosmetic surgery.

## Methods

### Ethics statement

This study was approved by the Medical Ethics Committee (No. [2009]58). Individuals attending our hospital for cosmetic surgery were asked to read information about the purpose of the study, and written informed consent was obtained from each participant.

### Data collection

An outpatient registration form was used to record the patient’s name, contact information, age, sex, marital status, occupational status, educational degree, and whether the patient had children. A total of 5010 patients attending our hospital for cosmetic surgery between January 2010 and December 2014 were asked to complete the outpatient registration form. Of these, 4550 patients agreed to fill out all the information; the remaining 460 refused to provide some of the information requested and therefore did not meet the inclusion criteria. The informational data were imported into Microsoft Excel 2007, and the data entry and collection were verified by two of the authors. The final data were analyzed manually using SPSS, version 20.0 (SPSS Inc., Chicago, IL, USA). Patient demographics were represented by the number 0, 1, 2 and 3. Further details are given in Table 1. Cosmetic surgery was converted to 0 or 1, respectively: 0 signifying no surgery, and 1 indicating surgery.

### Statistical analysis

Statistical analysis was performed using SPSS, version 20.0. Values are presented as the number and percentage. To determine independent predictive factors for the leading three cosmetic surgeries, we used logistic regression analysis. A *P* value of less than 0.05 was considered statistically significant.
